# Anti-oxidant and anti-apoptotic effects of royal jelly against polystyrene microplastic-induced testicular injury in mice

**DOI:** 10.22038/ijbms.2024.78787.17037

**Published:** 2024

**Authors:** Hojat Anbara, Maryam Ghorbani, Alireza Shahriary

**Affiliations:** 1 Department of Pharmacology and Toxicology, Faculty of Pharmacy, Baqiyatallah University of Medical Sciences, Tehran, Iran; 2 Chemical Injuries Research Center, Systems Biology and Poisonings Institute, Baqiyatallah University of Medical Sciences, Tehran, Iran

**Keywords:** Anti-oxidant, Apoptosis, Mitochondria, Polystyrene microplastic, Royal Jelly, Testicular toxicity

## Abstract

**Objective(s):**

In recent years, microplastics (MPs), which are novel environmental contaminants measuring 5 mm in diameter, have garnered considerable attention. However, information regarding substances that can mitigate the dangers of MPs for animals remains extremely limited.

**Materials and Methods:**

Ninety days were devoted to the exposure of mature male mice to royal jelly (RJ) and 2 µm virgin polystyrene microplastics (PS-MPs) in this study. Pre-implantation embryo development; the structure of testis tissue; the gonadosomatic index; sperm parameters; RNA damage in germinal cells; the anti-oxidant capacity of the entire testis; and the activity of anti-oxidant enzymes in serum and testicular tissue, including TAC, SOD dismutase, CAT, GSH, and MDA, histomorphometric indices of the testis (tubular differentiation index, spermatogenesis index, and repopulation index), steroidogenic foci, and the quantity of apoptosis were assessed in the testis, respectively, through the measurement of pro-apoptosis (p53, Bax, and Caspase-3) and anti-apoptosis (Bcl-2) factors, as well as Hsp70 mediator.

**Results:**

The results indicate that concurrent administration of RJ can confer a protective effect on mice exposed to microplastics by maintaining the structure of mitochondria and enhancement of the anti-oxidant defense system. Furthermore, RJ co-treatment decreased apoptosis and oxidant/anti-oxidant status, enhanced pre-implantation embryo development, and improved sperm characteristics and RNA damage in germ cells.

**Conclusion:**

The data confirm that royal jelly could protect the testis structure against polystyrene microplastic-induced testicular injury through anti-oxidant and anti-apoptotic properties.

## Introduction

Microplastics refer to plastic fragments that are less than 5 mm in length (1), and the pollution generated by them has received significant attention, and include polyethylene (PE), polypropylene (PP), polyvinyl chloride (PVC), polystyrene (PS) and polyethylene terephthalate (PET) (2). Plastics likely contaminate marine ecosystems worldwide, given their components are recently identified at depths ranging from around 7000 m to 10,890 m in the ocean. MPs released annually to terrestrial settings may be 4- to 23-fold more than that released to aquatic environments (3), they are frequently utilized in food packaging (4), building insulation (5), electrical equipment (6), cosmetics, and abrasive cleansers, etc. (7). In the environment, plastics may be fractured into fragments through various processes such as biodegradation, photodegradation, thermo-oxidative degradation, thermal degradation, and hydrolysis (8).

Due to their diminutive dimensions and sluggish degradation rate, MPs are readily ingested and accumulated by a variety of organisms in inland lakes (9), seawater (10), and even Polar Regions (11, 12). The majority of research assessing the toxicity of MP has focused on growth rate, nutrition rate, oxidative damage, egg-laying quantity, and enzyme activities (13, 14). Certain organisms, including the terrestrial isopod Porcellio scaber and the freshwater invertebrate Gammarus pulex, are not adversely affected by MPs, according to several studies (15, 16). A greater number of studies have demonstrated that MPs are an emergent threat to terrestrial ecosystems (17); therefore, it is necessary to have a more balanced dialogue regarding human exposure to MPs. In contrast to the greater scientific focus on the impacts of PMs on aquatic organisms, the effects of MPs on terrestrial systems have been relatively understudied (18, 19). In reality, the concentration of MPs in terrestrial ecosystems can range from four to twenty-three times that of the ocean (20). Furthermore, MPs have the potential to impact human health via the food chain, as they can be consumed by humans via bivalves, chicken gizzards, marine salt, and tap water (21, 22). PS-MPs are a commonly utilized copolymer in a wide range of disposable plastic items and packaging materials (23). The monomers of certain types of MPs, like polyethylene and polypropylene, are not typically present in aquatic environments. Nevertheless, it is noteworthy that the presence of PS-MP monomers and oligomers can be observed within these media (24). The accumulation of PS-MP particles occurs in the gills, intestines, and liver of organisms. These particles are introduced into the organism through ingestion and inhalation and subsequently enter the liver via the bloodstream (25). PS-MPs have been found to decrease testosterone synthesis and inhibit the activity of succinate dehydrogenase (SDH) and lactate dehydrogenase (LDH) enzymes. Reproduction is one of the most sensitive mechanisms. Current research is examining the detrimental impacts of MPs on the reproductive systems of aquatic organisms. Multiple investigations have uncovered a variety of harmful effects. Moreover, PS-MPs have the potential to increase ROS generation in oyster sperm cells (14). Limited research has been conducted on the effects of PS-MPs on the reproductive systems of mammals; however, it has been established that PS-MPs can disrupt the systems of males. The p38 MAPK pathway can be activated by PS-MPs, leading to impairment of sperm function and damage to testicular tissues (19). Hormonal concentrations and the expression of steroidogenic enzymes were both diminished by PS-MPs. In addition, the apoptotic profile was changed, and a decrease in the number of germ cells was noticed in testicular tissues in rats (22). However, it has been observed that the administration of anti-oxidant products and modulators of inflammatory effects can potentially mitigate these adverse effects (26).

For decades, bee products have been utilized in the treatment of a wide range of human ailments (27). Major Royal Jelly Proteins (MRJPs), which are well-known bioactive components of Royal Jelly (RJ), are regarded as a potential factor in extending honeybee life due to their exceptional biological properties (28, 29). An examination of RJ proteins revealed that 82–90% (w/w) is composed of MJRPs, and RJ contains complex proteins of the MRJP family and certain free amino acids that are vital for the nutrition of both the queen bee and larvae (30). “Bee Milk” is another name for fresh royal jelly, a pale yellow substance secreted by the mandibular and hypopharyngeal salivary glands of juvenile nurse Apis mellifera between the ages of 5 and 14 (29, 31). Globally, RJ is incorporated into the diet due to its advantageous nutritive, anti-oxidant, protective, and anti-inflammatory properties (32). RJ is composed of an extensive array of compounds, such as amino acids and nutrients (33). RJ contains a considerable quantity of exogenous amino acids, including valine, isoleucine, and leucine that have been branched (34). Additionally, it contains vitamins, minerals, and phenolic organic substances including flavonoids and phenolic acids, specific organic acids, and inorganic components (35). China currently holds the dual position of being the foremost producer and consumer of RJ globally (36). Several studies have indicated that RJ exhibits estrogenic properties comparable to exogenous steroid hormones such as testosterone and 17-estradiol (30). Extensive literature describes estrogen-like compounds that bear resemblance to estrogens and may modulate estrogen receptors (ERs) to induce a variety of estrogenic or anti-estrogenic effects in the reproductive systems. Exogenous estrogen, including estrogen-like compounds, is present in a variety of plant and animal-derived substances, including seeds, vegetables, milk, and dairy products (37). In the interim, it has been documented that RJ inhibits the adverse effects of exogenous estrogen on the male reproductive system (30). Additionally, prior research has demonstrated that RJ has the potential to improve sperm quality parameters, and testicular tissue structure, as well as reproductive toxicities reduction (28, 38).

Polystyrene is one of the most widely used plastics. In this study, we selected 2 μm PS-MPs as the test material to investigate whether PS-MPs cause testicular tissue damage in rats by studying the effect of PS-MP exposure on testicular structure and function. To determine the role of oxidative stress on the potential mechanism, royal jelly, a common anti-oxidant, was administered to prevent oxidative stress.

## Materials and Methods

***Chemicals and materials***

All used materials in the current study are as follows: the MPs were assigned from Sigma Co. (Sigma-Aldrich, Germany), acridine-orange staining dye was prepared from Pajohesh Asia Co. (Pajohesh Asia, Iran), human tubal fluid medium (HTF; CooperSurgical, USA) was obtained from Life-Teb-Gene Co. (Life-Teb-Gene, Iran), potassium simplex optimization medium (KSOM; Merck, Germany) was prepared from Elim-Teb Co. (Elim-Teb, Iran), and the pregnant mare serum gonadotropin (PMSG) and human chorionic gonadotropin (hCG) were from Sigma Co. (Sigma-Aldrich, Germany).

***Experimental design***

Thirty-six, pathogen-free, NMRI male mice (6 weeks old, 20 to 25 g in body weight) were purchased from Razi Vaccine and Serum Research Institute (Tehran, Iran) and accommodated in a specific standard facility with 22 ± 1 °C, a 12-hour light and dark cycle, and free access to standard food and water. International animal welfare guidelines and compatible local regulations for experimentation were followed during this study.

The mice were randomized to four groups (N = 9 for each group): the control group which received 0.10 ml ddH_2_O, the RJ group100 mg/kg, body weight (38), the PS-MPs group 1 mg/kg, body weight (39), and the RJ + PS-MPs group, administered by oral gavage once per day.

***Autopsy and sampling***

**Figure 1:**
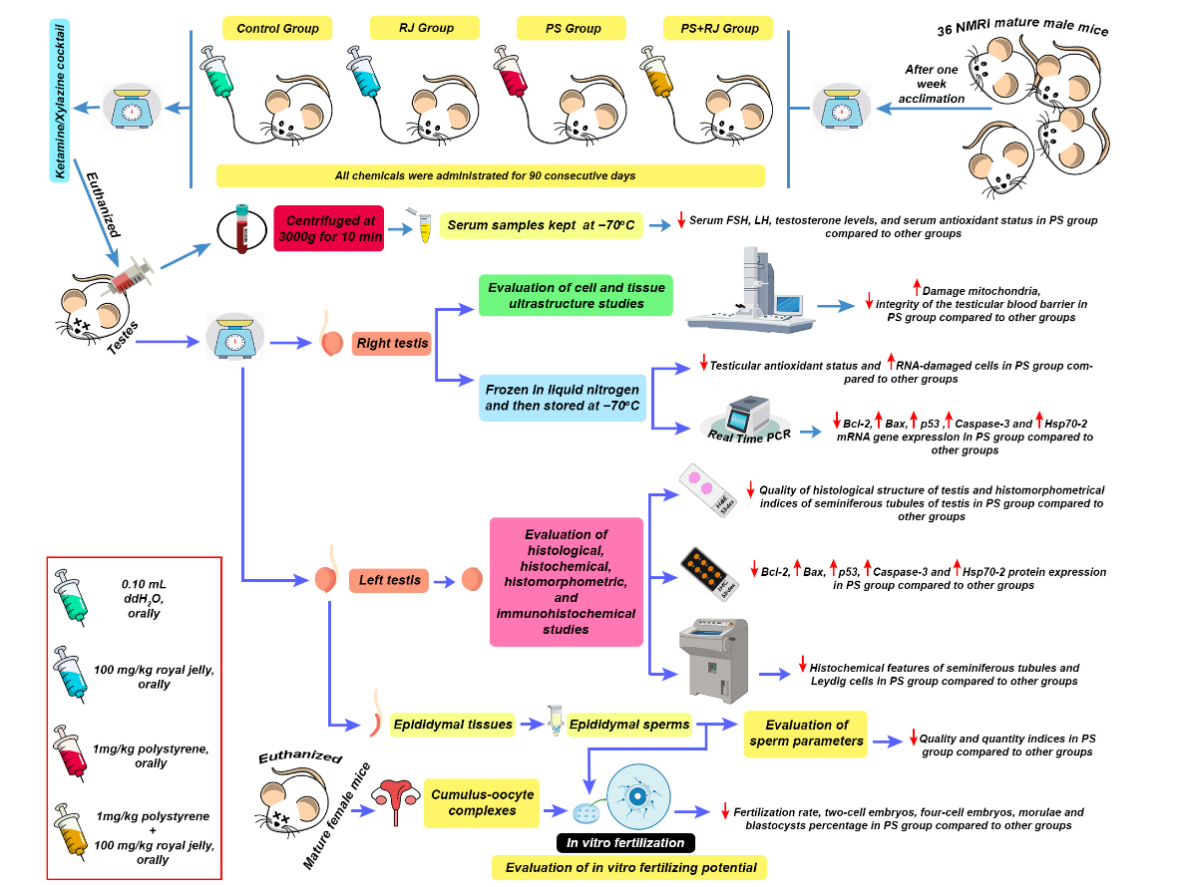
The graphical abstract of toxicity of polystyrene microplastics (PS-MPs) in male mice

Ninety days later, the blood sample was collected from the hearts of the mice, and animals were anesthetized by intraperitoneal injection of ketamine/xylazine cocktail (0.10 ml xylazine and 1 ml ketamine and 8.90 ml distilled water with the dose of 0.1 ml/10 g body weight), and gonads were weighed to determine the gonadosomatic index (GSI). Blood samples were obtained from lab tubes and centrifuged at 3,000g for 10 min. The resultant serum samples were frozen at −70 °C and preserved for later examination. The right testicle of each mouse was frozen in liquid nitrogen and then preserved at −70 °C until additional biochemical evaluations were done. The epididymis was washed with normal saline and sperm quality was detected. The left testicles of the animals were fixed in Bouin’s solution for histological examinations. GSI values were calculated by calculating the ratio of the weight of both testes to the body weight (Figure 1).

***Epididymal sperm quality***

Sperm count was assessed by the standard hemocytometer method. To investigate sperm DNA damage, the specimens were examined under a fluorescent microscope (Leitz, Germany). The percentages of spermatozoa with single-stranded DNA that fluoresced red, orange, or yellow relative to the total number of spermatozoa per sample were calculated and reported. The sperm abnormality percentage was determined using Papanicolaou staining. The aniline blue staining was examined for the detection of excessive histones in the process of sperm chromatin condensation. The percentage of motile sperm was also ascertained using the WHO standard method. Similarly, the eosin/nigrosin staining technique was utilized to determine the proportion of viable sperm (38).

***Sperm in vitro fertilizing potential***

After giving mature female mice a PMSG injection to stimulate superovulation, hCG (10 IU; injected intraperitoneally) was given 48 hr later. The animals were then euthanized after 14 hr. The oviducts were taken out and placed in a dish with a medium that had serum albumin [BSA] at a concentration of 4 mg/kg HTF added to it. Next, the cumulus-oocyte complexes that were collected were transferred. Fertile sperm (1×10^6^/ml HTF + 4 mg/kg BSA) were introduced. Fertilization was assessed by observing two pronuclei after 4–6 hr of incubation at 37 °C with 5% CO_2_. Subsequently, the zygotes were transferred to a new medium and cultivated for an additional 5 days to monitor early embryonic development.

***Histological analysis***

The fixed testis was embedded in paraffin, sectioned at 5-6 μm, stained with hematoxylin and eosin (H&E), and photographed under an optical microscope. ImageJ, Fiji software, and a Dino-Lite digital lens were utilized for histomorphometric analysis. Additionally, histometrical characteristics of the testes were assessed, such as the height of the germinal epithelium, the thickness of the testicular capsule, the diameter of the seminiferous tubules, and the quantity of Sertoli and Leydig cells. To categorize spermatogenesis, the criteria established by Johnsen were applied. The grading system for this classification utilizes tubule cross-sections containing graded scores ranging from 1 to 10, which indicate the presence or absence of primary cell types in a maturity-based order. Repopulation index, the quantity of spermatogonia with a heterochromatic nucleus, was separated into spermatogonia with an achromatic nucleus and represented as a percent (RI), the percentage of seminiferous tubules with three or more to tubules with less than three germinal epithelial cell layers was estimated as a tubal differentiation index (TDI), and the spermiogenesis index (SI) was conducted by calculating the percentage of seminiferous tubules with or without maturing sperm (40). Periodic acid-Schiff (PAS) staining was performed to determine carbohydrate deposition. Additionally, the frozen sectioning process was applied for histochemical evaluations. The samples were embedded using an optimal cutting temperature compound (OCT gel), and testicular tissue slices were cut at 15–20 m levels using a cryostat at -40 °C (SLEE, Germany). Sudan Black B (SB) staining is a histological technique used to demonstrate the presence of fats, lipids, triglycerides, and lipoproteins in tissue samples. This staining method is particularly useful for visualizing lipid droplets and fatty substances in tissues, such as the testis. It is especially valuable for identifying and measuring the cytoplasmic bio-steroid supplement in Leydig cells, and for assessing the rate of lipid foci supplementation in both treated and control animals. Alkaline phosphatase staining (ALP) was done to show how much of this enzyme is present as an inflammatory biomarker (40).

***Oxidative stress biomarkers***

Biomarkers of Oxidative stress in serum and tissue after homogenizing the frozen right testicles in ice-cold KCL (150 mM), were centrifuged at 3000g for 10 min. Following the collection of the supernatants, serum, and tissue samples were utilized to evaluate oxidative stress biomarkers. Utilizing the Benzie and Strain method for ferric reducing anti-oxidant power (FRAP), the total anti-oxidant capacity (TAC) was determined. The values were denoted, respectively, in nmol/ml of serum and nmol/mg protein testis tissue. To determine the rate of lipid peroxidation, the malondialdehyde (MDA) concentration of the samples in question was determined using the thiobarbituric acid (TBA) method, as outlined by Niehaus and Samuelsson. The MDA values were articulated. Serum and testis samples were analyzed for superoxide dismutase (SOD) activity using a SOD detection reagent Kono method by the manufacturer’s guidelines. The catalase (CAT) activity of the aforementioned samples was determined using the Koroluk method. The glutathione (GSH) activity in each sample was determined using a GSH detection reagent, Ellman, following the manufacturer’s instructions. Utilizing the Griess reaction, the total quantities of nitric oxide (NO) in the samples were determined (41).

***LH, FSH, and testosterone levels in serum***

LH and FSH concentrations in the serum were determined utilizing a radioimmunoassay kit (SimulTRAC, LH/FSH, MP Biomedicine, Hungary). Utilizing a competitive chemiluminescent immunoassay kit, testosterone levels were determined (DRG, Germany).

***Immunohistochemical staining***

In testicular tissue, immunohistochemical (IHC) staining was conducted by the protocol established by Anbara *et al*. to identify the presence of p53, Bcl-2, Bax, Caspase-3, and Hsp70-2 proteins. To accomplish this, tissue sections measuring 5-6 μm were prepared and subsequently subjected to oven heating at 60°C for 20 min (Venticell, MMM, Einrichtungen, Germany). Following deparaffinization (in xylene, 2X) and rehydration, the antigen was unmasked using a sodium citrate buffer (10 mM, pH = 7.2). Subsequently, the sample was incubated in a solution containing 0.03% hydrogen peroxide to inhibit endogenous peroxidase. Following a PBS wash, the transparencies were incubated overnight at 4 °C with primary antibodies (p53, Bcl-2, Bax, Caspase-3, and Hsp70-2). Next, the slides were rinsed with PBS and incubated in a humidified compartment for 10 min with anti-polyvalent antibody and HRP, respectively. Following a 10-second incubation with 3,3´-diaminobenzidine (DAB) substrate, the slides were counterstained with Harris hematoxylin. Following this, the slides were rinsed. The sections were subsequently dehydrated, xylene-cleared, and mounted. The sections that had undergone IHC staining were examined and assessed using a light microscope that was outfitted with a digital camera (Leica EC3, Germany). In five sections from each group, the number of positive cells per square millimeter of tissue was determined and compared across groups. Furthermore, the pixel-based frequency of the positive reactions was analyzed and contrasted between groups using Image-Pro Insight Software (version 9.00, Media Cybernetics) on 20 photomicrographs of cross-sections measuring 2530 μm × 2530 μm. 

***mRNA extraction***

In this study utilizing TRIZOL, the total mRNA content of the tissue samples was extracted. For this purpose, 20–30 mg of testicular tissue was homogenized. Upon extraction, the extracted RNAs exhibited an acceptable adsorption ratio of 280 to 260 nanometers, falling within the acceptable range of 1.8 to 2.0 adsorption. cDNA was produced by combining 1 μg of total mRNA with a reaction mixture of 20 volumes. The following were utilized in accordance with the manufacturer’s protocol: oligo (dT) primer (1 μl), 5 reaction buffer (4 μl), RNAse inhibitor (1 μl), 10 mM dNTP mix (2 μl), and M MuLV Reverse Transcriptase (1 μl). The researchers conducted a three-replicate PCR reaction utilizing a MyGo PCR mini thermal cycler (USA). A volume of 0.5 μl (approximately 5–10 ng) of cDNA template was combined with 10 μl of 2 × SYBR GREEN master mix (PCRbio, Cat No. PB20.12) in the qPCR reaction combinators. The 2^–∆∆Ct ^Ct method was employed to compute the relative quantification values of the target genes, with GAPDH serving as the internal reference (Table 1).

***Testicular RNA damage analysis***

Analysis of RNA damage was conducted as previously detailed (41). To identify testicular germ cells harboring damaged RNA, RNA loss and/or the presence of diffuse red-colored RNA were utilized. To identify the intact cells, vibrant red RNA was detected at the apex of the nucleolus.

***Evaluation of steroidogenic foci in leydig cells***

Leydig cells were analyzed with the histogenotech Lab (Tehran, Iran) fluorescent reagent specifically designed for intracytoplasmic steroid droplets. In summary, the cryo microtome (SLEE, Germany) was utilized to section the specimens, and the dehydration procedure was executed using a descending series of ethanol. Following a 5-minute hematoxylin staining period, the slides were counterstained with a special fluorescent dye designed for steroids (FITC-conjugated 1-an ilinonaphthalene-8-sulfonate). Subsequently, the slides were rinsed under flowing water. Following a rinse with distilled water, the transparencies were then covered. The quantification of Leydig cells per square millimeter of testicular interstitial tissue was performed.

***TUNEL/DAPI staining***

Following deparaffinization, the transparencies are rinsed with PBS (P4417; Sigma), and the sample is then immersed in a solution of methanol and H_2_O_2_ (7722-84-1; Sigma) for 10 min. The samples then undergo three washes with PBS, each lasting five minutes. Subsequently, the sample is treated with proteinase K (21627M; Sigma) at 37 °C for 30 min. Following three PBS rinses, 0.3% Triton is added to the sample to increase the permeability of the nucleus; the solution is left to sit for ten minutes. The samples are subjected to three PBS washes. The sample is treated with Roche TdT (11684817910) at 37 °C for two hours before being rinsed three times in PBS. The DAPI-stained nuclei were subsequently transferred to the coverslip in preparation for fluorescent microscopy with an Olympus microscope. Furthermore, the quantification of apoptotic cells was conducted. The TUNEL-positive cell rate is proportional to the ratio of apoptotic cells to total cells in each visual field.

***Electron microscopy***

After isolating cellular tissue specimens, they were immersed in a fixative solution containing 2.5% glutaraldehyde and 1% osmium tetroxide (MERCK, Germany). Throughout the tissue monitoring process, (OsO4) was utilized. Testicular sections, measuring 80 nm in thickness, were sectioned onto copper grids after dehydration of the tissues using an acetone series and immersion in Araldite (CY212, TAAB, E006, UK) to facilitate polymerization. Photographs of the sections were taken subsequent to their contrasting with uranyl acetate and lead citrate using a Zeiss EM 900 (42).

***Statistical analysis***

In order to conduct statistical analyses, SPSS software (Version 16.00, USA) was utilized. The Mann–Whitney test was utilized to compare the relative quantities of mRNA across all categories. Comparative analysis of histological, biochemical, oxidative stress biomarkers, and *in vitro* fertilization (IVF) data across all groups utilized was one-way ANOVA followed by the Tukey post-hoc test. A significance level of *P*-value below 0.05 was employed, and all data were presented as the mean ± standard error.

## Results

***Effects of PS-MPs and RJ on BWA and GSI***

Body weight alternation (BWA) showed no significant difference (*P*>0.05) groups compared to the control group. As shown in Table 2, the administration of PS-MPs had a significant (*P*<0.05) decrease compared to the control and RJ groups, and in the PS-MPs plus RJ group, there was a significant (*P*<0.05) increase compared to the PS-MPs group, but there was a significant (*P*<0.05) decrease in the two control and RJ groups (Table 2)**.**

***Effects of PS-MPs and RJ on FSH, LH, and testosterone hormones***

The administration of PS-MPs significantly (*P*<0.05) decreased the serum concentration of FSH, LH, and testosterone compared to the control group, on the other hand, receiving PS-MPs+RJ caused a significant increase compared to the PS-MPs group (Table 2).

***Effects of PS-MPs and RJ on OS biomarkers***

According to the data presented in Table 2, the serum and testicular TAC levels were significantly (*P*<0.05) decreased by PS-MPs, compared to the control and RJ animals. Also, there was a significant increase in the serum of PS-MPs+ RJ compared to PS-MPs. The level of SOD activity in the serum and testicular tissue of mice treated with PS-MPs was significantly reduced compared to other prescribed groups (*P*<0.05). CAT activity decreased significantly (*P*<0.05) in the PS-MPs group compared to other groups. The GSH activity was significantly decreased (*P*<0.05) when PS-MPs were administered in comparison to the control group. Also, there was a non-significant (*P*>0.05) increase in the PS-MPs+ RJ group compared to PS-MPs. Moreover, the NO and MDA contents of animals administered PS-MPs were significantly increased (*P*<0.05) in comparison to the control group. Also, in the PS-MPs+ RJ group, compared to PS-MPs, there was a significant decrease (*P*<0.05) and this increase was moderated to some extent (Table 2).

***Effects of PS-MPs and RJ on sperm quality***

**Figure 2:**
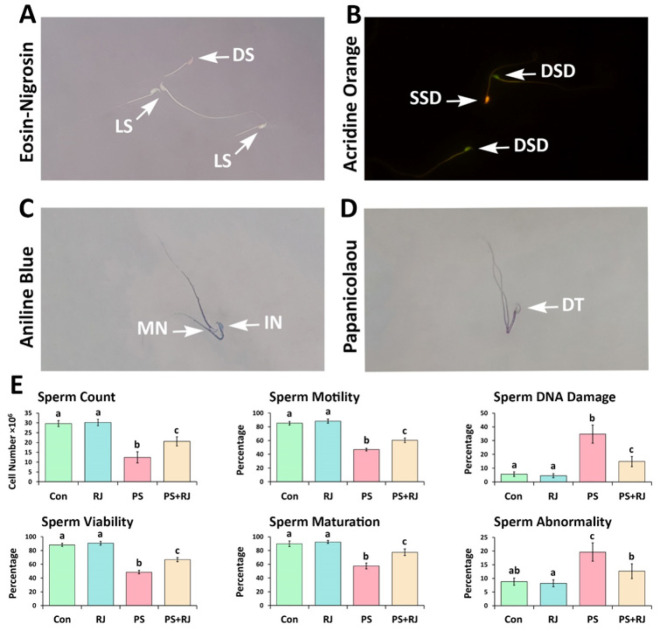
Photographs of epididymal spermatozoa in mice

The PS-MPs group exhibited a significant decrease in sperm count, motility, DNA damage, viability, and maturation as determined by observations (*P*<0.05). On the other hand, simultaneous use of RJ significantly improved the decrease in sperm quantity and quality caused by PS-MPs (*P*<0.05). Furthermore, RJ significantly reduced sperm abnormality induced by PS-MP consumption (*P*<0.05). There were no significant differences (*P*>0.05) observed in sperm parameters when comparing the control group to the group that received RJ alone (Figure 2).

***Sperm in vitro fertilizing capacity***

**Figure 3:**
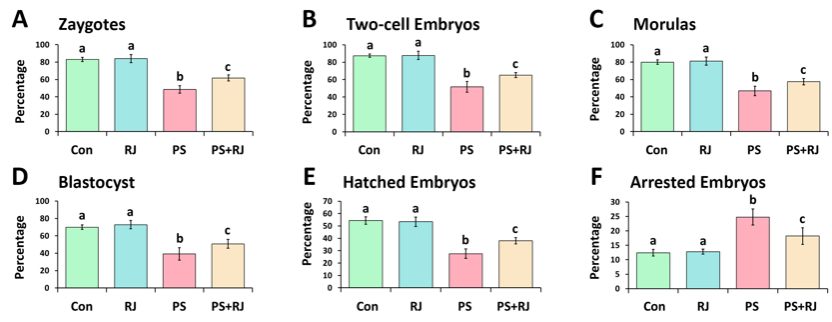
Effects of PS-MPs and royal jelly on* in vitro* fertilization outcome in male mice

The findings revealed a significant decrease in the reproductive capacity of the sperm in the treated PS-MPs group (*P*<0.05). Nonetheless, co-administration of RJ significantly ameliorated the effect of PS-MPs, and increased sperm fertilizing potential (*P*<0.05). As a result, simultaneous treatment of RJ and PS-MPs resulted in increased pre-implantation embryo development compared to the treated group (Figure 3).

***Effects of PS-MPs and RJ on histomorphometric indices***

**Figure 4:**
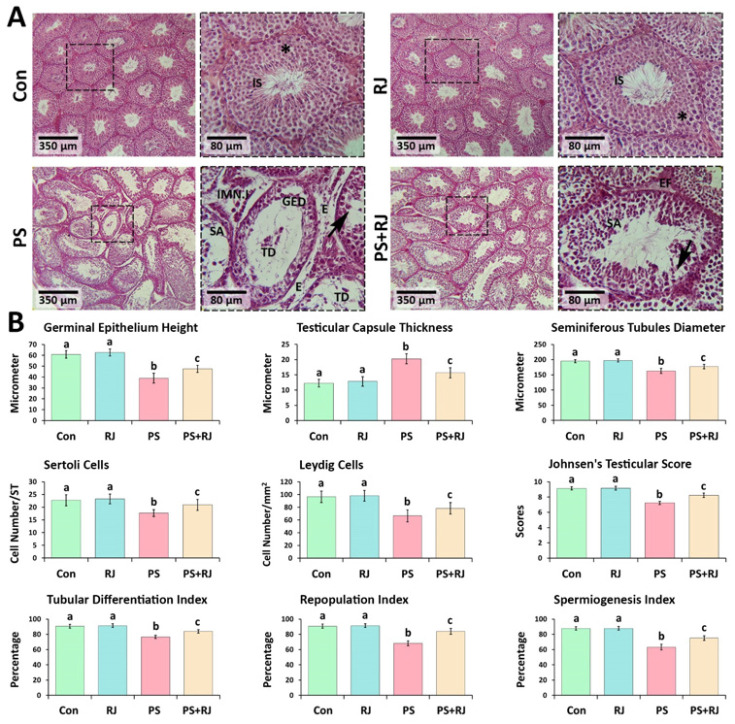
Hematoxylin and eosin; cross sections from intact and damaged seminiferous tubules

As shown in Figure 4 A, based on our histological observations, it was determined that PS-MPs could induce severe edema and disarrayment in connective tissue. An elevation in tubular depletion and germinal epithelium dissociation (GED) was noted in the group treated with PS-MPs. The administration of PS-MPs was particularly associated with significant morphological alterations in the testes. Some seminiferous tubules had undergone atrophy, which signified a substantial decline in the number of germ cells, intense infiltration of immune cells, accumulation of edematous fluid, and widening of the intertubular space within interstitial connective tissue. In the treatment group with PS-MPs + RJ, all the mentioned negative effects improved. 

The percentages of seminiferous tubules exhibiting positive TDI, SPI, and RI were significantly (*P*<0.05) reduced in PS-MPs-treated animals, in comparison to the control group. Furthermore, when compared to the control group, the germinal epithelium height, testicular capsule thickness, seminiferous tubule diameter, Johnsens testicular score, and the number of Sertoli and Leydig cells, were significantly reduced (*P*<0.05) in animals treated with PS-MPs. However, the effects of PS-MPs and RJ led to a statistically significant difference (*P*<0.05) with the PS-MPs group (Figure 4 B).

**Figure 5:**
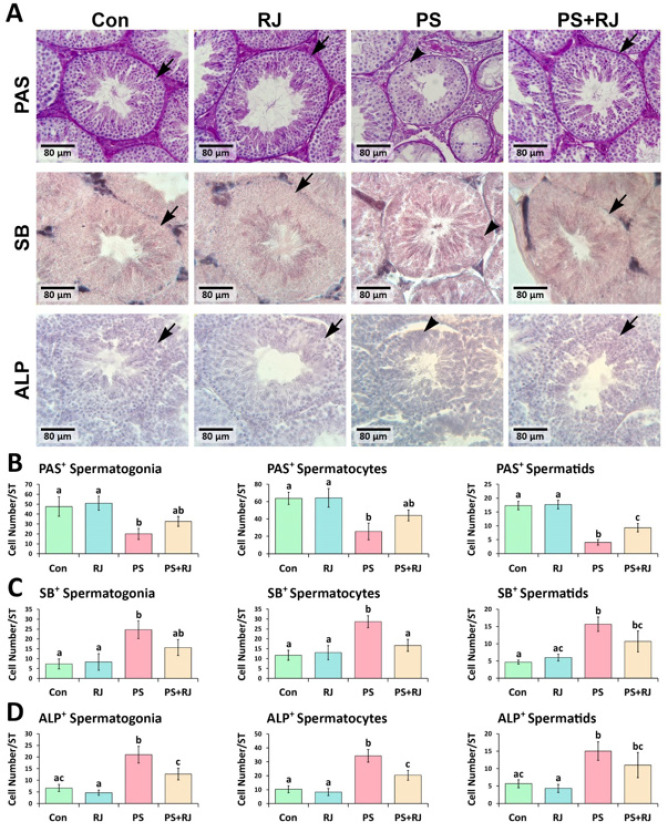
(A) Periodic acid Schiff (PAS), Sudan-black B (SB), and Alkaline phosphatase (ALP) staining of the cross-sections from experimental groups; physiological intra-cytoplasmic lipid and carbohydrate storage in the germ cells are presented with arrows and the changed pattern of storage is marked with arrowheads. (B) Mean distribution of PAS+ cells, (C) SB+ cells, and (D) ALP+ cells in each seminiferous tubule in the PS-MPs group vs the other groups. Values represent mean ± SD (N=9). Different superscripts indicate significant differences (*P*<0.05) between groups.

To examine the impact of PS-MPs and RJ on the storage of carbohydrates, lipids, and alkaline phosphatase, intra-cytoplasmically in the germ cells, the PAS, SB, and ALP staining methods were employed. The PS-MPs-treated group exhibited a statistically significant increase (*P*<0.05) in the average number of SB^+^ and ALP^+^ spermatogonia, spermatocytes, and Spermatid per seminiferous tubule. This significant increase (*P*<0.05) was less in the PS-MPs+ RJ group than in the PS-MPs group. Furthermore, it was observed that the PS-MPs group exhibited a significantly lower mean count of PAS^+^ spermatogonia, spermatocytes, and Spermatid cells (*P*<0.05) in comparison to the control and RJ mice. In the group treated with PS-MPs+ RJ, these values increased significantly (*P*<0.05) compared to the PS-MPs group (Figure 5A), consequently, the software was used to analyze the overall changes in a cross-section using a photomicrograph. Photomicrographs were analyzed using software. The results show a significant decrease (Figure 5B) in the intensity of red reactions indicating carbohydrate content, a significant increase in mean pixel-based intensities of black reactions indicating lipid foci (Figure 5C), and an increase in brown foci indicating ALP accumulation (Figure 5D), compared with control mice (*P*<0.05).

***Effects of PS-MPs and RJ on mRNA expression of p53, Bcl-2,*** ***Bax, Caspase-3, and Hsp70-2***

**Figure 6:**
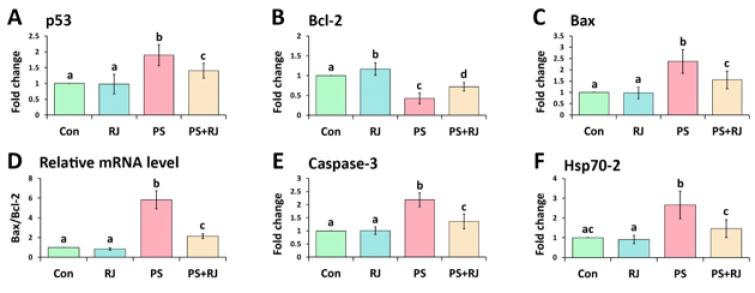
Relative mRNA levels of (A) p53, (B) Bcl-2, (C) Bax, (D) Bax/Bcl-2, (E) Caspase-3, and (F) Hsp70-2 in mouse testes were detected with q-PCR by normalizing to GAPDH. Values represent mean ± SD (N=9). Different superscripts indicate significant differences (*P*<0.05) between groups.

By utilizing qRT-PCR, the expression of p53, Bcl-2, Bax, Caspase-3, and Hsp70-2 mRNA in the testis was assessed (Figure 6). The results of the qRT-PCR analysis indicated that the relative mRNA expression of p53 (Figure 6A) was significantly (*P*<0.05) increased when PS-MPs were administered in comparison to the control group. In the PS-MPs+ RJ receiving group in comparison with the PS-MPs group, there was a significant decrease (*P*<0.05). Furthermore, the mRNA expression of Bcl-2 was significantly (*P*<0.05) increased by RJ, however, it significantly decreased (*P*<0.05) by PS-MPs compared to the control group (Figure 6B). Also, there was a significant increase (*P*<0.05) in the group treated with PS-MPs+ RJ compared to the PS-MPs group.

The mRNA expression of Bax (Figure 6C) differed, no significant difference (*P*>0.05) was observed in the group treated with RJ and the control group, on the other hand, there was a significant increase (*P*<0.05) in the PS-MPs treated group compared to the control group, and a significant decrease (*P*<0.05) in the PS-MPs+ RJ group compared to the PS-MPs treated group. 

Examining the expression ratio of Bax/Bcl-2 (Figure 6D) in the group treated with PS-MPs showed a significant increase (*P*<0.05) compared to the control group and the RJ group, on the other hand, it led to a significant decrease (*P*<0.05) in the group treated with PS-MPs+ RJ compared to PS-MPs group. The mRNA expression of Caspase-3 was found to be significantly increased (*P*<0.05) in animals treated with PS-MPs compared to the control and RJ groups (Figure 6E), however, it significantly decreased (*P*<0.05) in the group treated with PS-MPs+ RJ compared to the PS-MPs group. The mRNA expression of Hsp70-2 was found to be significantly increased (*P*<0.05) in animals treated with PS-MPs compared to the control and RJ groups (Figure 6F), however, it significantly decreased (*P*<0.05) in the group treated with PS-MPs+ RJ compared to the PS-MPs group.

***Effects of PS-MPs and RJ on protein expression of p53, Bcl-2, Bax, Caspase-3, and Hsp70-2***

**Figure 7:**
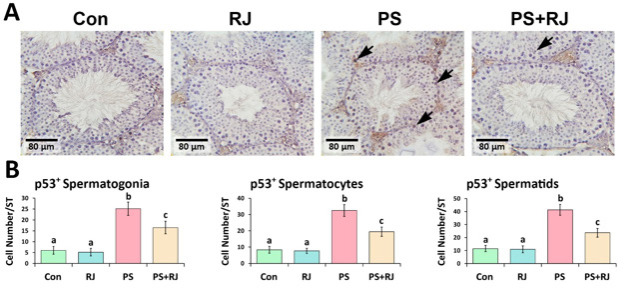
(A) Immunohistochemical staining of p53: The positive cells are marked with arrows. Increased positive germ cell distribution per one seminiferous tubule in the cross-sections of experimental groups. (B) Mean number of Bcl-2 positive germ cells per seminiferous tubule in different groups. Values represent mean ± SD (N=9). Different superscripts indicate significant differences (*P*<0.05) between groups.

The IHC staining of p53, Caspase-3, Bax, and Hsp70-2 protein expression significantly increased (*P*<0.05) in p53 (Figure 7), Bax (Figure 9), Caspase-3 (Figure 10), and Hsp70-2 (Figure 11) germ cells in the cross-sections of the PS-MPs received group compared to the control and RJ group. Accordingly, the mean distributions of p53, Bax, Caspase-3, and Hsp70-2 positive spermatogonia, spermatocytes, and spermatids were increased (*P*<0.05) in the PS-MPs received group compared to the control group mice. On the other hand, these values significantly decreased in the group receiving PS-MPs+ RJ compared to the PS-MPs group. 

**Figure 8:**
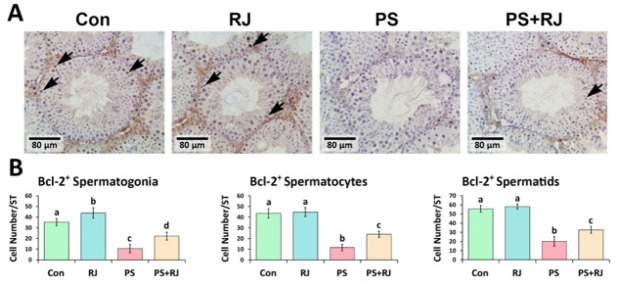
(A) Immunohistochemical staining of Bcl-2: The positive cells are marked with arrows. Increased positive germ cell distribution per one seminiferous tubule in the cross-sections of experimental groups. (B) Mean number of Bcl-2 positive germ cells per seminiferous tubule in different groups. Values represent mean ± SD (N=9). Different superscripts indicate significant differences (*P*<0.05) between groups.

Furthermore, the mean distribution of Bcl-2 positive (Figure 8) spermatogonia, spermatocytes, and spermatids in the PS-MPs receiving group was significantly decreased compared to the control mice and significantly increased in the PS-MPs + RJ treatment group. Software analysis exhibited a similar result. The photomicrographs have shown a significant (*P*<0.05) reduction in the mean pixel-based intensities of brown reactions representing p53, Bcl-2, Bax, Caspase-3, and Hsp70-2 reactions per cross-section.

***Effects of PS-MPs and RJ on apoptosis at germ cell***

Regarding alterations in redox biomarkers, DNA fragmentation, and mRNA damage, TUNEL and special fluorescent staining techniques were used to track ROS-induced harm in testicular tissue. The findings indicated that PS-MPs increased the number of apoptotic cells in the seminiferous tubules according to the same criteria observed in other groups (Figure 12.A.D). Consequently, the group that received PS-MPs exhibited the greatest abundance of apoptotic spermatogonia, spermatocytes, and spermatids relative to total cells in the seminiferous tubules (Figure 12.A.D). On the other hand, the group that received PS-MPs+RJ showed less apoptotic spermatogonia, spermatocytes, and spermatids in the seminiferous tubules than the PS-MPs group, as determined by the same criteria per seminiferous tubules (Figure 12.A.D). The purpose of the specific fluorescent analyses was to evaluate the accumulation of steroid foci within the cytoplasm of Leydig cells. As shown in Figure 12.B.E, long-term administration of PS-MPs significantly (*P*<0.05) decreased the ratio of steroid foci in Leydig cells. Also, simultaneous treatment with RJ significantly increased (*P*<0.05) the steroid focus (Figure 12.B.E).

Analogous to its impact on the quantity of cellular DNA, PS-MPs significantly increased (*P*<0.05) the damage to mRNA in the testicular tissue (Figure 12.C.F). The red fluorescent reactions, representing intact mRNA content, were significantly reduced (*P*<0.05) in the cross-sections of mice administered PS-MPs compared to the RJ receiving group of mice (Figure 12.C.F). In the group treated with RJ, the red fluorescent reactions were significantly (*P*<0.05) higher than in the group of mice receiving PS-MPs (Figure 12.C.F).

***Effects of PS-MPs and RJ on mitochondrial ultrastructure***

Examination of the ultrastructure of the mitochondria cells of the seminiferous tubules in the control and RJ groups does not have any abnormality. In the PS-MPs treatment groups, mitochondria ultrastructure was seriously damaged, this damage was reduced in the PS-MPs+ RJ treatment group compared to the PS-MPs group (Figure 13.B).

Con: Control; RJ: Royal jelly; PS: Polystyrene

**Table 1:** Forward (F) and reverse (R) primer sequences of the gene of interest for qRT-PCR

Genes	Forward	Reverse
Caspase-3	GTTAACACGAGTGAGGATGTG	TACCCTGAAATGGGCTTGTGT
Bax	TGGCGATGAACTGGACAACAAC	CCCGAAGTAGGAAAGGAGGC
Bcl-2	CTGGTGGACAACATCGCTCTG	GGTCTGCTGACCTCACTTGTG
p53	GACTTCTTGTAGATGGCCATGG	ATGGAGGAGTCACAGTCGGATA
Hsp70-2	CAGCGAGGCTGACAAGAAGAA	GGAGATGACCTCCTGGCACT
GAPDH	GCAAGAGAGAGGCCCTCAG	TGTGAGGGAGATGCTCAGTG

**Table 2:** Effect of different doses of polystyrene on weight parameters, serum parameters, and biomarkers of oxidative stress in different groups of mice

Parameters	Con	RJ	PS	PS+RJ
BWA (g)	8.33±1.19^a^	8.25±0.86^a^	7.82±1.06^a^	8.07±0.82^a^
Gonadosomatic index	0.72±0.02^a^	0.72±0.02^a^	0.61±0.03^b^	0.65±0.02^c^
FSH (mIU/ml)	0.26±0.01^a^	0.27±0.01^a^	0.15±0.01^b^	0.20±0.01^c^
LH (mIU /ml)	0.27±0.01^a^	0.29±0.01^a^	0.15±0.01^b^	0.23±0.01^c^
Testosterone (ng/ml)	8.17±0.38^a^	4.36±0.40^b^	6.29±0.41^c^	7.91±0.34^a^
Serum TAC (µmol/L)	137.26±3.65^a^	143.91±4.28^a^	94.57±4.08^b^	115.99±4.94^c^
Serum SOD (U/L)	1.201±0.058^a^	0.577±0.064^b^	0.907±0.070^c^	1.136±0.068^a^
Serum CAT (U/L)	11.88±0.36^a^	6.79±0.31^b^	8.69±0.34^c^	11.57±0.47^a^
Serum GSH (U/L)	1.53±0.39^a^	0.63±0.20^b^	0.86±0.26^b^	1.50±0.21^a^
Serum MDA (µmol/L)	1.93±0.18^a^	6.94±0.33^b^	4.72±0.26^c^	2.15±0.17^a^
Serum NO (µmol/L)	35.01±4.09^a^	81.59±6.34^b^	58.81±5.03^c^	37.44±3.20^a^
Tissue TAC (nmol/mg)	1.19±0.10^a^	0.65±0.08^b^	0.80±0.11^b^	1.10±0.12^a^
Tissue SOD (U/mg)	21.12±0.95^b^	15.85±1.06^c^	18.09±0.79^a^	19.31±0.85^a^
Tissue CAT (U/mg)	1.16±0.09^a^	0.69±0.10^b^	0.86±0.13^b^	1.17±0.09^a^
Tissue GSH (U/mg)	3.67±0.46^b^	1.37±0.73^c^	2.29±0.62^ac^	3.04±0.66^ab^
Tissue MDA (nmol/mg)	4.61±0.39^a^	10.18±0.72^b^	7.98±0.69^c^	4.76±0.41^a^
Tissue NO (nmol/mg)	0.46±0.06^a^	1.15±0.09^b^	0.72±0.07^c^	0.48±0.06^a^

All data are presented as Mean ± SD

Con: Control, RJ: Royal jelly, PS: Polystyrene. BWA: Body weight alternations; FSH: Follicles stimulating hormone; LH: Luteinizing hormone; TAC: Total antioxidant capacity; SOD: Superoxide dismutase; CAT: Catalase; GSH: Glutathione; MDA: Malondialdehyde; NO: Nitric oxide. Values represent mean ± SD (N=9). Different superscripts in the same row show significant differences between groups (*P*<0.05).

(A) Eosin-nigrosin staining, (B) Acridine-orange, (C) Aniline-blue, and (D) Papanicolaou, each at a magnification of 1,000X. LS; Live sperm. DS; dead sperm. MN; Mature nucleus. IN; immature nucleus. SSD; Singel-strand DNA. DSD; Double-strand DNA, and DT; double-tail. (E) Mean sperm quality parameters in the PS- MPs group vs the other groups. Values represent mean ± SD (N=9). Different superscripts indicate significant differences (*P*<0.05) between groups.

Percentage of (A) zygotes, (B) two-cell embryos, (C) morulas, (D) blastocyst, (E) hatched embryos, and (F) arrested embryos in different groups. Values represent mean ± SD (N=9). Different superscripts indicate significant differences (*P*<0.05) between groups.

(A) Cross sections from experimental groups present reduced epithelial height as well as germinal epithelium dissociation (GED), edema (E) and oedematous fluid accumulation (EF), immune cells infiltration (IMN.I), atrophic and depletion seminiferous tubules (TD), intact spermatogenesis (IS), Spermatogenesis arrest (SA), Germ cells integrity (*), and germ cells dissociation (arrows) of interstitial connective tissue. (B) Mean histomorphometric alternation in the PS-MPs group vs the other groups. Values represent mean ± SD (N=9). Different superscripts indicate significant differences (*P*<0.05) between groups.

**Figure 9:**
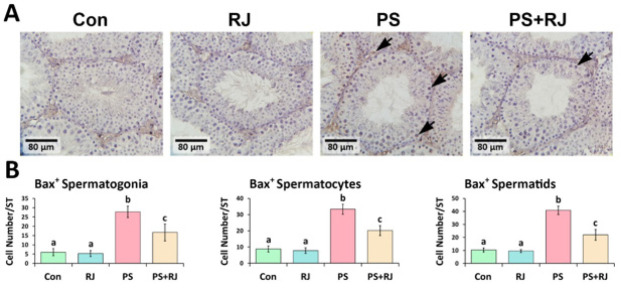
(A) Immunohistochemical staining of Bax: The positive cells are marked with arrows. Increased positive germ cell distribution per one seminiferous tubule in the cross-sections of experimental groups. (B) Mean number of Bax-positive germ cells per seminiferous tubule in different groups. Values represent mean ± SD (N=9). Different superscripts indicate significant differences (*P*<0.05) between groups.

**Figure 10:**
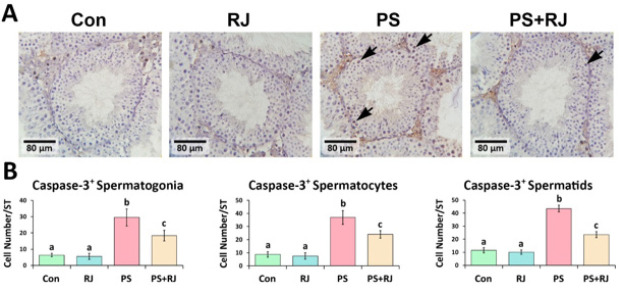
(A) Immunohistochemical staining of Caspase-3: The positive cells are marked with arrows. Increased positive germ cell distribution per one seminiferous tubule in the cross-sections of experimental groups. (B) Mean number of Caspase-3 positive germ cells per seminiferous tubule in different groups. Values represent mean ± SD (N=9). Different superscripts indicate significant differences (*P*<0.05) between groups.

**Figure 11:**
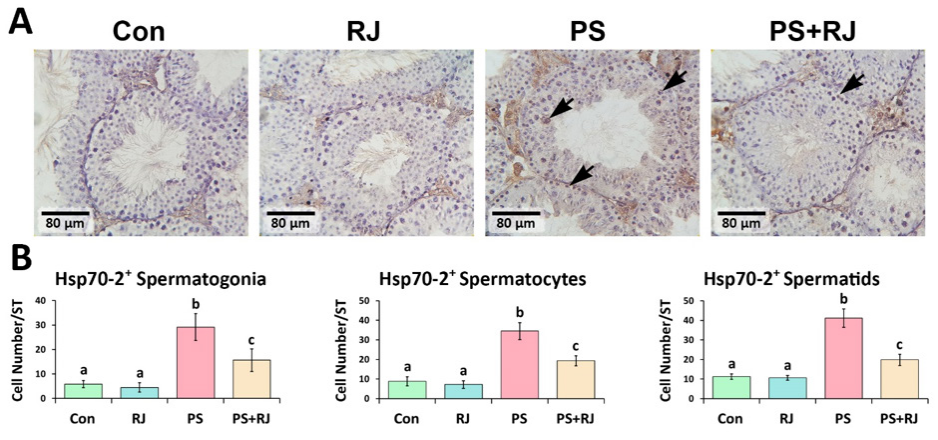
(A) Immunohistochemical staining of Hsp70-2: The positive cells are marked with arrows. Increased positive germ cell distribution per one seminiferous tubule in the cross-sections of experimental groups. (B) The mean number of Hsp70-2 positive germ cells per seminiferous tubule in different groups. Values represent mean ± SD (N=9). Different superscripts indicate significant differences (*P*<0.05) between groups.

**Figure 12:**
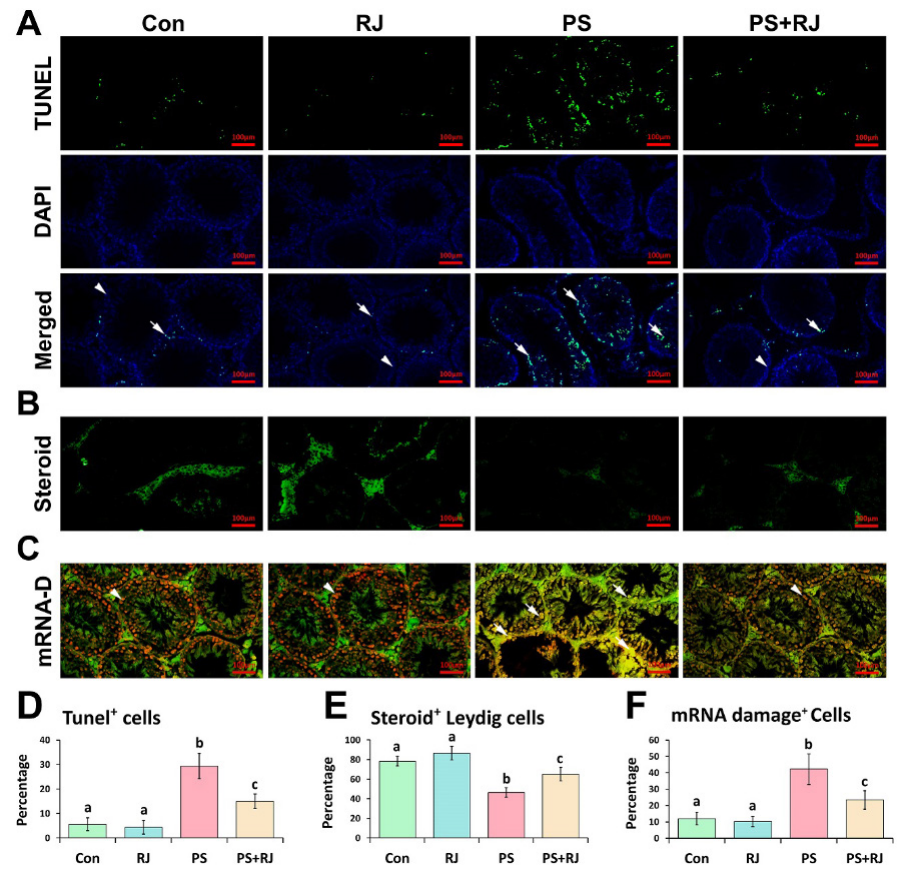
(A) TUNEL-DAPI technique; the head arrows represent cells with normal mRNA and DNA while the arrows represent DNA and mRNA damage. Percentage of Tunel-positive cells per seminiferous tubules of testicular tissue. (B) Intracytoplasmic steroid-specific fluorescent staining for Leydig cells in interstitial connective tissue; the proportion of steroid-positive Leydig cells per mm^2^ of interstitial tissue. (C) mRNA-damage technique; the head arrows represent cells with normal mRNA while the arrows represent mRNA-damage. Percentage of mRNA-damage cells per seminiferous tubules of testicular tissue. Values represent mean ± SD (N=9)

Different superscripts indicate significant differences (*P*<0.05) between groups.

**Figure 13:**
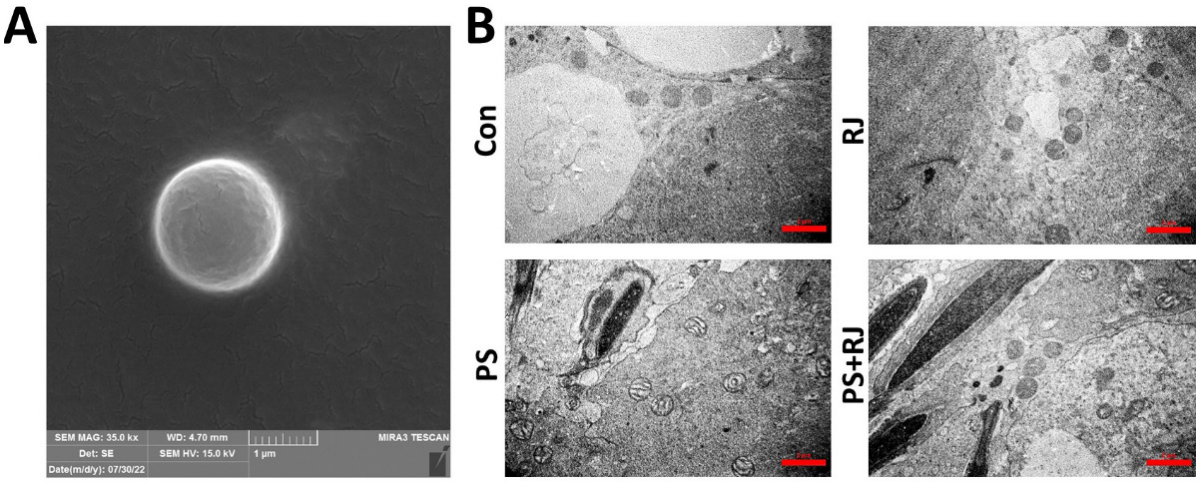
(A) Morphological characterizations of 2 µm PS-MPs

Scanning electron microscope (SEM) photomicrographs showing the size and shape of PS-MPs. (B) The mitochondrial structure was disrupted by PS-MP treatment. Ultrastructural changes of mitochondrial structure induced by PS-MPs and RJ were examined by an electron microscope. (Scale bar: 2 μm).

## Discussion

This research aimed to examine the efficacy of royal jelly in reducing oxidative stress, induced by polystyrene microplastic in the mice testes. Toxicity was demonstrated by PS-MPs, which induced different changes in histological and biochemical parameters in experimental mice testis tissue (43, 44). The present results showed a high deficit in the group treated with PS-MPs alone, compared to the control, royal gel group, and PS-MPs group treated with royal gel in seminal fluid parameters, including sperm motility, and more disorders and gave morphological normality, concentration, and reduction of sperm density in the lumen of the spermatogenic tube. In addition, the histopathological results showed severe necrosis, atrophy, and abnormal organization of spermatogenic tubules in the testes of the PS-MPs group (39, 45). The toxic agents created in this study bind to free radicals that are the main initiators of testicular lipid peroxidation (46, 47). Testes consumed glutathione as a result of increased lipid peroxidation after exposure to PS-MPs (48). The expression of Caspase-3 was increased in the testes after exposure to PS-MPs (48).

Hormone imbalances were observed in the groups that received PS-MPs (49). As a result of its anti-oxidant activity and ability to prevent oxidation of plasma lipids, RJ was demonstrated to substantially modify the experimental groups. Mammalian research has shown that RJ increases testosterone levels in mice (50, 51). Until now, the effectiveness of RJ extract as a preventive treatment against PS-MP toxicity has not been studied. A previous report showed the deleterious effect of PS-MPs on the lipid profile of mice. 

PS-MPs were associated with considerable alterations in histological, hormonal, and semen parameters, according to this study. Seminiferous tubules improved in the group that received RJ, and spermatogenesis appeared normally, with spermatozoa present in the lumen of the seminiferous tubules (52). RJ demonstrated an ameliorative impact on the testes of diabetic mice in a prior investigation. Furthermore, the enhancement in spermatogenesis is apparent through a substantial increase in the quantity of cells at various spermatogonial stages, Sertoli cells, and interstitial Leydig cells, in comparison to control negative mice (53). However, RJ is utilized due to its higher concentration of bioactive compounds, which confers anti-oxidant properties (54).

According to the findings of the present investigation, PS-MPs cause severe testicular damage. The group that did receive PS-MPs demonstrated significant tubular atrophy, edema, and a reduced proportion of tubules displaying positive TDI, RI, and SPI. Moreover, PS-MPs induced significant RNA degradation in the germinal epithelium of the testis and a decline in sperm quality. Co-administration of RJ subsequent to PS-MP induction, on the other hand, substantially mitigated the PS-MPs-induced aberrations by enhancing testicular anti-oxidant and endocrine functions. Excessive documentation exists regarding the physiological functions that reactive oxygen species (ROS) expression at optimal levels performs, including sperm capacitation, viability, and DNA integrity. The oxidative stress that is produced has the potential to induce significant harm to lipids, DNA, and proteins, ultimately resulting in the apoptosis or necrosis of living cells (55, 56). 

PS-MPs reduced sperm count, motility, and viability and increased chromatin de-condensation and DNA damage (45). Oxidative stress-induced lipid peroxidation increases sperm cell membrane permeability, causing sperm mortality (19). Impaired sperm maturation causes improper chromatin condensation, which increases DNA fragmentation in sperm (38). Thus, PS-MPs-induced oxidative stress reduces sperm quality by affecting spermatogenesis and spermiogenesis (45). Using RJ as an anti-oxidant chemical increased sperm quality (38) by reducing PS-MPs-induced oxidative stress.

Previous animal models show that sperm DNA damage is linked to poor embryo development (57). Lower progressive motility and sperm morphology can slow pre-implantation embryo development to the blastocyst stage (58). In line with that, IVF was performed to determine how PS-MPs affect sperm *in vitro* fertilizing potential and pre-implantation embryo development and how RJ improves PS-MPs-reduced IVF ratio (59). We tried to reveal the role of Caspas-3, Bax, Bcl-2, p53, and HSP70 as genes involved in apoptosis, if apoptosis is reduced, increased sperm quality can improve IVF results.

Biochemical evaluation of PS-MP administration in male rats showed changes in hormonal concentrations. PS-MPs reduce lower luteinizing hormone (LH), follicle-stimulating hormone (FSH), plasma testosterone, and testicular testosterone (22). PS-MPs disrupt testosterone secretion and destroy the testicular blood barrier and testicular inflammation, which can cause various damages to the sperm. Also, by facilitating the penetration of PS-MP particles, these particles accumulate in the testicular tissue and disrupt the tissue structure of mice testes (60). In the current study, concomitant administration of RJ to PS-MPs receiving mice remarkably improved all PS-MPs-induced negative changes in the hormonal concentrations. The protection offered by RJ against PS-MP reproductive toxicity is likely thanks to its ability to reduce oxidative stress through neutralizing ROS as well as PS-MPs-induced RNA damage reduction (38, 61).

The Tunel experiment indicated that long-term PS-MP use, especially at high doses, may promote animal cell death by apoptosis. Toxins harm cells and cause apoptosis. Oxidative stress helps cause apoptosis. PS-MPs caused anti-oxidant status in PS-MPs-treated animals and OS-induced apoptosis. As a result, mitochondria are exposed to damage due to oxidative stress. Sperm and testis DNA and mRNA damage increased in this study. In the testis, Bcl-2 was down-regulated and p53, Bax, and Caspase-3 were up-regulated at gene and protein levels. Apoptosis involves these factors. p53 induces proapoptotic protein transcription after DNA damage, promoting apoptosis. Bcl-2, an antiapoptotic mediator, controls caspase proteases and mitochondrial cytochrome c release (62). In contrast, Bax activation releases cytochrome c into the cytosol (63). Cellular morphology during apoptosis is controlled by caspase-3 (64). Apoptosis is induced by PS-MP ingestion by down-regulating antiapoptotic mediators and up-regulating apoptotic mediators due to mRNA and DNA damage (65). By reducing the amount of oxidative stress caused by the consumption of microplastics, RJ can reduce the amount of cell apoptosis (66) caused by the administration of PS-MPs. This work is consistent with others that have found PS-MP consumption down-regulates antiapoptotic mediators and up-regulates apoptotic mediators in diverse organs of experimental animals. PS-MP consumption compromises the integrity of the mitochondrial membrane and induces oxidative stress. Additionally, PS-MPs have the potential to cause cellular dysfunctions, including an increase in the expression of Hsp70-2 and the distribution of certain proteins; severe damage to DNA and homeostasis components, including chaperones; and severe oxidative stress (67, 68). 

## Conclusion

The present study showcased the efficacy of RJ in mitigating the oxidative stress induced by PS-MPs in a mouse model of testis inflammation. This was confirmed by the improvements observed in semen parameters such as concentration, motility, vitality, and morphological normalcy, in addition to the histopathological structure of seminiferous tubules. The levels of all male reproductive hormones, including testosterone, LH, and FSH, have shown a notable reversal, offering promise for natural products with potent anti-oxidative and therapeutic properties. These products include semen and sex hormone improvers. Moreover, RJ reduced the mitochondria-dependent apoptosis at the germ cell level, improved expression levels of Hsp70-2, and promoted embryo development. However, more clinical studies are necessary to investigate the potential protective effects of long-term consumption of RJ on the male reproductive system under the influence of PS-MPs in humans.
